# Relapse Following Allogeneic Hematopoietic Cell Transplantation for Acute Myeloid Leukemia Apparently Due to Somatic Cell Evolution via Epigenetic Variation and Immune Selection

**DOI:** 10.20411/pai.v4i1.285

**Published:** 2019-02-27

**Authors:** Neil S. Greenspan

**Affiliations:** Case Western Reserve University, Cleveland, Ohio

**Keywords:** acute myeloid leukemia (AML), malignancy, blasts, allogeneic hematopoietic cell transplantation (HCT), chemotherapy, relapse, T cells, cytotoxicity, whole exome sequencing, mutation, epigenetic, HLA class II, HLA class I, interferon gamma, clone, subclone, fitness, somatic cell evolution

## Abstract

In this brief commentary, I discuss a recently published study that documents the role of immune escape in relapse of acute myeloid leukemia (AML) after hematopoietic cell transplantation (HCT). Of particular interest, the mechanism identified by the authors for the ability of the malignant cells to evade destruction by host T cells is the loss of cell surface expression of HLA class II molecules based on processes other than mutation. The authors labeled this mechanism for altered cell surface display of HLA class II antigens “epigenetic.”

This study should be of strong interest for immunologists, oncologists and even specialists in infectious diseases for several reasons. First, the results extend the range of examples for which epigenetic mechanisms can play a critical role in resistance to therapy in oncology or infectious disease. Second, findings relating to decreased cell surface display of HLA class II molecules motivate investigation of novel approaches using cytokines to increase the numbers of HLA class II proteins on malignant myeloid cell membranes and reduce the extent of immune escape by these cells. Third, the data presented suggest experimental directions intended to clarify detailed molecular mechanisms underlying the cases of AML post-HCT relapse and raise questions relating to why some mechanisms of somatic cell evolution and not others are operative in different clinical settings.

## BACKGROUND

Acute myeloid leukemia (AML) is a hematopoietic malignancy with an average five-year survival of less than 30% [1]. Relapse is common after standard chemotherapy. Allogeneic transplantation with hematopoietic stem (and other) cells (HCT), that is, transfer of such cells from a donor to a genetically non-identical recipient, can be effective in extending life in some patients who have AML. However, relapse following this form of treatment is also common.

In previous work, Tim Ley and his colleagues at Washington University School of Medicine in St. Louis [2] and other groups sought to determine the mechanisms underlying relapse after drug treatment. They used extensive genomic DNA sequencing of serial samples of AML cells from patients to demonstrate that relapse after chemotherapy is often associated with new somatic mutations that confer fitness advantages on subclones of malignant myeloid cells. The cells that harbor these mutations are better able to resist the therapeutic drugs and over time their progeny come to dominate the malignant myeloid cell population. Thus, the failure of chemotherapy is often attributable to undesirable somatic cell evolution.

### Key Finding: An Unexpected Basis for Immune Escape and Relapse

In new work, Ley, John DiPersio, and colleagues studied AML patients who received hematopoietic cell transplants from HLA-matched related, HLA-matched unrelated, or HLA-mismatched unrelated donors but not haploidentical donors. They reasoned that relapse after allogeneic HCT in these settings might be due, as was seen for relapse after chemotherapy, to the occurrence of somatic mutations that create fitter subclones able to “take over” the population of malignant myeloid cells [3]. However, extensive whole exome sequencing of AML blasts from many patients who had relapsed post-transplant as well as non-malignant control cells (skin or T cells) from these same patients failed to reveal recurring somatic mutations in the malignant cells that might account for the development of fitter subclones. Consistent with this interpretation of the sequencing results, the range of mutations seen in cells from patients with post-transplant relapse were not significantly different from the mutations in cells from patients with post-drug therapy relapse. If changes in nucleotide sequence were responsible for immune escape post-transplant, recurrent changes in genes different from the genes frequently mutated in post-drug therapy relapse would have been expected because the mechanisms of escape are presumed to be different in these two therapeutic contexts.

Prior studies of AML patients revealed that, in some cases of allogeneic HCT with cells from HLA-matched donors, relapse was associated with loss of HLA genes. In some patients exhibiting relapse after HCT from haploidentical donors (ie, donors who share one set of HLA alleles with the recipient but differ for the second set of HLA alleles on the other chromosome 6) a subset of AML cells delete the portion of the chromosome containing the non-shared HLA alleles. The whole exome sequencing results in the paper by Christopher *et al.* revealed [3] a deletion in the HLA region for only one of 15 patients who had suffered relapse following HCT. In this case, the deletion did not affect coding sequences, which was unlike the results with haploidentical transplants in other studies.

### Evidence for Epigenetic Variation Underlying Immune Escape

The authors tested the notion that relapse was driven by subclones with altered patterns of transcription that somehow conferred greater fitness. Total RNA sequencing was performed on seven patients with relapse post-HCT. For purposes of comparison, similar testing was performed on nine patients with relapse after chemotherapy. Given the authors' prospective cutoffs for significant differences in levels of messenger RNA, for the post-HCT patients, 187 genes were found to have decreased transcription and 34 genes had increased amounts of corresponding cellular mRNA. AML cells from the patients with relapse after drug therapy exhibited only eight genes with significant changes in expression. For the post-transplant patients, many of the genes with altered levels of RNA transcripts were related to immunological pathways, including HLA class II genes.

In six of the seven patients studied, the HLA class II loci with significantly decreased expression in AML cells included HLA-DPA1, HLA-DPB1, HLA-DQB1, and HLA-DRB1. Additional loci encoding other proteins involved in antigen processing and presentation by HLA class II molecules were also found to have significantly reduced expression after relapse. Using flow cytometry, the authors verified that post-relapse AML cells displayed fewer cell surface class II proteins than matched pre-relapse AML cells, using a fluorescently-tagged antibody able to recognize HLA-DR, -DQ, and -DP proteins.

Treatment of the AML cells from patients with post-transplant relapse with interferon-gamma, a cytokine known to increase expression of HLA class II genes, was able to increase the number of class II molecules on cell surfaces of AML cells as assessed by flow cytometry. Functional immunological assays based on T-cell activation were consistent with these flow cytometric results.

### Therapeutic Implications

These findings raise the possibility that therapies designed to restore pre-relapse levels of production of class II HLA proteins could be useful in the context of preventing relapse post-HCT. Of course, it is fair to wonder if such therapy would completely solve the problem of immune escape or if additional genetic or epigenetic variations would undermine the approach.

The authors looked for subclones with low class II gene expression among the AML blasts at presentation but found none. They therefore argue that, after HCT, malignant cells that “randomly” reduced cell surface expression of HLA class II molecules evaded destruction by CD4+ T cells with greater probability than cells that maintained relatively higher levels of class II molecule display. Cells with decreased cell surface class II proteins caused relapse. The authors' reasonable conclusion is that the underlying mechanism for immune escape is epigenetic.

### Technical Caveat

An important limitation pertaining to the authors' interpretation of what they call enhanced exome sequencing is that mutations in non-coding portions of the genomes of the AML cells were not assessed and could have contributed to immune escape while eluding detection by this type of analysis. In principle, such mutations could influence the binding of transcription factors to control regions of loci relevant for immune escape or the activities of other genes or their gene products (proteins or RNA molecules) that influence either transcription or downstream processes that are required for the production of, for example, HLA class II molecules. If such mechanisms were involved in the decreased cell surface display of class II proteins on AML blast cell surfaces, it would arguably be fair to regard the mechanism of immune escape as both genetic and epigenetic, where the latter term refers to changes in gene transcription or subsequent steps leading to gene product synthesis and appropriate cell trafficking of these gene products.

### Unanswered Questions and Future Research

Many intriguing questions are raised by the findings of Christopher *et al.*, such as why decreased expression of HLA class II genes is sufficient for relapse despite continued expression of class I genes at “normal” levels. Given the continued expression of class I HLA genes and the display of class I molecules on the surfaces of malignant myeloid cells, it would be of interest to know if CD8+ T cells can eliminate these cells in some patients.

Related to the above, I would like to know what mechanism or mechanisms are used by T cells to destroy the AML cells. The most obvious possible mechanisms include direct cytotoxicity based on perforin and granzymes or effects based on cytokines secreted by T cells. Both CD4+ and CD8+ T cells can potentially perform either or both functions.

Another aspect of post-HCT relapse in need of further clarification is the detailed nature of the molecular processes responsible for the decreased production and cell surface display of class II HLA proteins. Assuming the authors are correct in their conclusion that the reduction of class II molecules on the malignant cell plasma membranes is epigenetic and not genetic, why is that the case? While there are a variety of potential mutations in the coding or non-coding regions of the various class II loci that could reduce the amount of gene product for one locus, there may not exist mutations—other than deletions—directly affecting these particular loci, that would affect production of all of the major class II gene products. However, mutations in the coding or non-coding regions of the key transcription factors for class II loci might be able to mediate a global reduction in HLA class II antigens on AML cell surfaces. It is also fair to wonder, as indirectly alluded to above, whether immune escape could involve either epigenetic, genetic, or both mechanisms in some patients. Future larger-scale investigations, involving more patients from multiple medical centers, should potentially reveal whether there are additional mechanisms of post-HCT immune escape, including typical types of changes in nucleotide sequence.

The authors did not identify the precise molecular mechanism or mechanisms responsible for the decreased display of class II HLA molecules on the AML cells of their patients who relapsed post-HCT. I expect that many specialists in HCT would like to know if the reductions in class II molecule production and cell surface display are based solely on alterations of transcription rates or if other processes could contribute. Examples of other possibly relevant processes include regulation of: 1) mRNA half-life; 2) translation efficiency; 3) protein half-life; or 4) some combination of these. It would also be of interest to know whether the relevant variations in any of these mechanisms from cell to cell are just random alterations that are favored by immune selection. If so, one can ask how they gain a degree of stability. Whether such changes can be further stabilized by actual changes of nucleotide sequence in the class II genes themselves or at other loci that encode gene products involved in the processes that transform RNA transcripts into cell surface gene products would also be useful to clarify. At least some of these interesting and possibly clinically relevant questions are likely to be addressed in future studies.

### Broader Implications

The findings reported by Christopher *et al.* provide another example of the potential for cellular selection based on phenotypic variation that is not strictly “genetic” (ie caused by changes in the nucleotide sequences of genes or other stretches of DNA) to affect health, disease pathogenesis, or response to treatment. In other words, cellular selection based on phenotypic variation that is due to changes in gene transcription or in subsequent steps of the process by which genetic information is transformed into gene products (sometimes referred to as “epigenetic”), can strongly influence clinical outcomes. There are reports demonstrating that this form of “non-genetic” cellular evolution can lead to therapeutic failure for bacteria treated with antibiotics [4] and for tumor cells treated with chemotherapeutic agents [5]. Therefore, insights into why, in the patients followed in this study, post-HCT relapse in AML is due primarily to epigenetic and not genetic mechanisms could be of interest and relevance to physicians treating cancers other than AML and to clinicians treating infections in which drug-resistant pathogens threaten patient health and survival.
